# Cold evoked potentials elicited by rapid cooling of the skin in young and elderly healthy individuals

**DOI:** 10.1038/s41598-022-07967-x

**Published:** 2022-03-09

**Authors:** Paulina Simonne Scheuren, Natascha Nauer, Jan Rosner, Armin Curt, Michèle Hubli

**Affiliations:** 1 Spinal Cord Injury Center, Balgrist University Hospital, University of Zurich, Forchstrasse 340, 8008 Zurich, Switzerland; 2 Department of Neurology, University Hospital Bern, Inselspital, University of Bern, Bern, Switzerland

**Keywords:** Pain, Sensory processing, Somatosensory system, Chronic pain, Neurological disorders

## Abstract

Cold-evoked potentials (CEPs) constitute a novel electrophysiological tool to assess cold-specific alterations in somatosensory function. As an important step towards the clinical implementation of CEPs as a diagnostic tool, we evaluated the feasibility and reliability of CEPs in response to rapid cooling of the skin (−300 °C/s) and different stimulation sites in young and elderly healthy individuals. Time-locked electroencephalographic responses were recorded from at vertex in fifteen young (20–40 years) and sixteen elderly (50–70 years), individuals in response to 15 rapid cold stimuli (−300 °C/s) applied to the skin of the hand dorsum, palm, and foot dorsum. High CEP proportions were shown for young individuals at all sites (hand dorsum/palm: 100% and foot: 79%) and elderly individuals after stimulation of the hand dorsum (81%) and palm (63%), but not the foot (44%). Depending on the age group and stimulation site, test–retest reliability was “poor” to “substantial” for N2P2 amplitudes and N2 latencies. Rapid cooling of the skin enables the recording of reliable CEPs in young individuals. In elderly individuals, CEP recordings were only robust after stimulation of the hand, but particularly challenging after stimulation of the foot. Further improvements in stimulation paradigms are warranted to introduce CEPs for clinical diagnostics.

## Introduction

Neuropathic pain is a debilitating sequela of a wide range of conditions due to a lesion or disease of the somatosensory system^1^. Patients with neuropathic pain report varying signs and symptoms, including sensory loss and hypersensitivities to different stimulus modalities^2^. Cold allodynia is commonly reported in patients with neuropathic pain^3,4^, particularly in small fiber neuropathy^5,6^, postherpetic neuralgia, and acute oxaliplatin-induced polyneuropathy^7^. Moreover, loss of cold sensation and cold allodynia is often present in patients with neuropathic pain after spinal cord injury^8^ and central post-stroke pain^9,10^. Cold deficits have also been reported in 60% of patients with polyneuropathy and loss of cold detection is predominant in the early stage of Fabry’s disease due to small fiber pathology^11,12^. While quantitative sensory testing allows the detection of cold-specific alterations in somatosensory function^3,13^, this method remains subjective and relies heavily on the individuals compliance. Clinical neurophysiological tools, i.e., laser- and contact heat evoked potentials (LEPs and CHEPs), have emerged as valid measures for the assessment of spinothalamic pathways in peripheral and central neurological disorders^14^. However, cold evoked potentials (CEPs) could complement the diagnostic work-up of patients with neuropathic pain, particularly those presenting with cold-specific abnormalities. Notably, CEPs may allow the objective characterization of alterations in cutaneous cold-mediating primary afferents and sub-modality specific spinothalamic pathways^15–17^. While the recording of CEPs was already introduced in the 1970s^18,19^, the implementation of CEPs into the clinical routine has since been lagging behind that of CHEPs and LEPs. In comparison to heating ramps used for the assessment of CHEPs (70 °C/s) or LEPs, the limited steepness of cooling ramps (i.e., −20 °C/s) produces less robust brain signals with limited reliability^20,21^. To overcome this issue, a novel device integrated with micro-Peltier elements has been developed through which rapid cooling of the skin can now be achieved (i.e., up to 300 °C/s)^22^. These methodological advances in terms of cold stimulators have fostered the ability to record robust CEPs with a high signal-to-noise ratio and latencies comparable to the conduction velocity of A-delta fibers in healthy young individuals^22–24^. Furthermore, this cold stimulator was useful to detect cold specific damage to small fibers and within central pathways in two patients with cold hypoesthesia and allodynia, respectively^23^. These previous investigations demonstrated the robustness of recording CEPs with steep cooling ramps in healthy individuals, yet none of them included elderly individuals in their study cohorts. The feasibility of CEPs in healthy elderly individuals is indispensable, as this population is representative of the clinically important patient population under investigation. In addition, no studies have yet explored the usefulness of rapid-cooling ramps for the recording of CEPs after stimulation of the feet, which are often the initial areas affected by length-dependent small fiber pathology^12^. Lastly, improved acquisition of CEPs after stimulation of glabrous skin would offer novel insights into the usefulness of CEPs to assess palmar symptoms, which may dominate the clinical phenotype in certain painful neuropathies^7^. Therefore, improved feasibility and reliability of CEPs across different age groups, skin types, and from distal body parts is a steppingstone towards the clinical implementation of CEPs.

The aim of the present study was to investigate the robustness of CEPs across different age groups (i.e., young and elderly) in response to rapid cooling of three clinically meaningful body areas (i.e., hand dorsum, hand palm, and foot dorsum). We hypothesized that rapid cooling of the skin would lead to improved acquisition and reliable CEPs for all age groups and stimulation areas.

## Results

### Individuals

A total of 31 individuals participated in both test I and test II. The young group consisted of eight females and six males (mean age 26 ± 4.5 years) and the elderly group of ten females and six males (mean age 57.9 ± 6.6 years). The time between test I and II was 14.7 ± 3.6 days. The neurological examination excluded any indication of subclinical changes due to polyneuropathy in all elderly individuals. QST revealed normal thermal thresholds after stimulation of the hand dorsum (CDT = 30.7 ± 0.6 °C; WDT = 34.8 ± 2.2 °C; CPT = 10.4 ± 8.7 °C; HPT = 44.4 ± 3.2 °C) and the foot dorsum (CDT = 28.6 ± 1.9 °C; WDT = 41.4 ± 3.7 °C; CPT = 9.0 ± 8.7 °C; HPT = 46.2 ± 5.3 °C) in the elderly individuals.

### CEP acquisition

**Figure 1:**
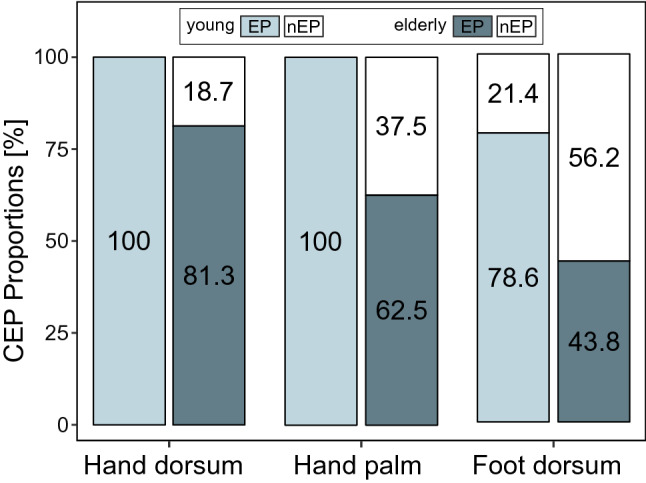
CEP proportions. Percent of present (EP) and absent (nEP) CEPs after stimulation of the hand dorsum, hand palm, and foot dorsum for the young and elderly age group.

At test I, CEP waveforms were identified in 90.3% across all individuals (young and elderly) at the hand dorsum (N = 28/31), 80.6% at the hand palm (N = 25/31), and only 60.0% at the foot dorsum (N = 18/30). One young individual presented with missing data at test I after stimulation of the foot dorsum due to technical signal artefacts (i.e., high noise level). CEP proportions for each age group are shown in Fig. 1. CEP acquisition was more challenging in the elderly age group, especially after stimulation of the foot dorsum (Fig. 1).

### Differences in CEP parameters between stimulation sites

**Figure 2:**
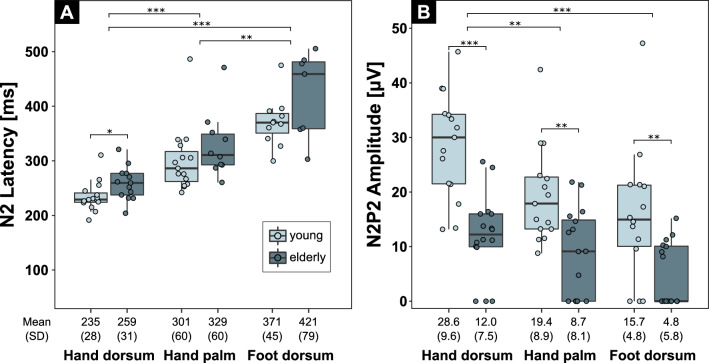
Comparison of cold evoked potential parameters between stimulation sites and age groups. The differences in N2 latencies **(A)** and N2P2 amplitudes **(B)** between the three stimulation sites (i.e., hand dorsum, hand palm, and foot dorsum) are shown for all individuals. The differences in CEP parameters between the young and elderly group are shown for each stimulation site. Statistical significance: **p < .01; ***p < .001.

N2 latencies differed significantly between stimulation sites (X^2^(2) = 36.5, p < 0.001) (see Fig. 2A for mean ± sd) (Fig. 2A). N2 latencies were shorter after stimulation of the hand dorsum compared to that of both the hand palm (W = 0, p < 0.001) and the foot (W = 0, p < 0.001). N2 latencies were also shorter after stimulation of the hand palm compared to that of the foot (W = 15, p < 0.01).

N2P2 amplitudes also differed significantly between stimulation sites (X^2^(2) = 18.5, p < 0.001) (Fig. 2B). N2P2 amplitudes were higher after stimulation of the hand dorsum compared to that of the hand palm (W = 378, p < 0.01) and the foot (W = 363, p < 0.001). N2P2 amplitudes did not differ between the hand palm and foot (W = 235, p = 0.15).

The cold stimulation induced a painless cold sensation in all individuals and ratings did not differ between stimulation sites after multiple comparisons (p > 0.05 for all comparisons). Cold ratings for test I can be found in Table 1.

**Table 1:** Test–retest analyses.

	Age group	Test I (mean ± sd)	Test II (mean ± sd)	ICC (95% CI)	Bland–Altman coefficients (mean ± 1.96 SD)	N
**A. Hand dorsum**						
N2 latency (ms)	Young	235 ± 28	248 ± 39	0.35 (−0.14 to 0.72)	−12.9 ± 74.3	15
	Elderly	260 ± 35	278 ± 71	0.59 (0.03–0.88)	−18.0 ± 98.0	10
N2P2 amplitude (μV)	Young	28.6 ± 9.6	27.8 ± 10.9	0.89 (0.70–0.96)	0.7 ± 9.8	15
	Elderly	13.7 ± 6.3	10.7 ± 7.0	0.59 (0.13–0.85)	3.0 ± 11.1	14
Cold Rating (NRS)	Young	2.5 ± 1.1	2.4 ± 1.2	0.85 (0.60–0.95)	0.1 ± 1.2	15
	Elderly	2.2 ± 0.9	2.4 ± 1.4	0.62 (0.19–0.85)	−0.2 ± 2.0	16
**B. Hand palm**						
N2 latency (ms)	Young	290 ± 33	289 ± 21	0.29 (−0.34 to 0.72)	1.2 ± 65.2	13
	Elderly	331 ± 63	317 ± 73	0.91 (0.65–0.98)	14.4 ± 54.3	9
N2P2 amplitude (μV)	Young	19.4 ± 8.9	16.1 ± 8.0	0.45 (−0.02 to 0.77)	3.3 ± 17.1	15
	Elderly	11.5 ± 7.2	10.7 ± 5.0	0.41 (−0.22 to 0.79)	0.8 ± 13.5	12
Cold rating (NRS)	Young	2.5 ± 1.1	2.6 ± 1.1	0.87 (0.65–0.96)	−0.17 ± 1.1	15
	Elderly	2.8 ± 1.3	2.8 ± 1.3	0.85 (0.63–0.95)	0.1 ± 1.4	16
**C. Foot dorsum**						
N2 latency [ms]	Young	371 ± 45	357 ± 59	0.53 (−0.05 to 0.85)	13.9 ± 99.9	11
	Elderly	452 ± 64	449 ± 71	−0.69 (−1.85 to 0.77)	3.8 ± 225.4	4
N2P2 amplitude [μV]	Young	20.0 ± 10.4	19.6 ± 7.1	0.89 (0.63–0.97)	0.4 ± 8.6	11
	Elderly	9.5 ± 4.4	6.5 ± 6.2	0.23 (−0.42 to 0.77)	3.0 ± 12.9	8
Cold Rating (NRS)	Young	2.1 ± 0.8	2.0 ± 1.0	0.71 (0.25–0.91)	0.1 ± 1.4	14
	Elderly	2.0 ± 1.2	2.1 ± 1.3	0.81 (0.53–0.93)	−0.1 ± 1.6	16

Mean ± standard deviation (sd) for N2 latencies, N2P2 amplitudes, and cold ratings. Test–retest statistics are shown as intraclass correlation coefficients (ICC) with 95% confidence interval (CI) (“poor” < 0.40, “fair” = 0.41–0.60, “moderate” = 0.61–0.80, and “substantial” = 0.81–1.00) and Bland–Altman coefficients (test I–test II; mean ± 1.96 standard deviation (SD), limit of agreement) for all CEP parameters, stimulation sites (i.e., hand dorsum, hand palm, and foot dorsum), and age groups (i.e., young and elderly).

*N* number of individuals, *NRS* numeric rating scale (0 = ‘not cold’ to 10 = ‘most imaginable cold’).

### Differences in CEP parameters between age groups

N2 latencies were significantly shorter in the young compared to the elderly group after stimulation of the hand dorsum (W = 149, p < 0.05) (Fig. 2A). There was, however, no difference in N2 latencies between age groups after stimulation of the hand palm or the foot.

N2P2 amplitudes were significantly higher in the young compared to the elderly group after stimulation of the hand dorsum (W = 19, p < 0.001), hand palm (W = 48, p < 0.01), and foot (W = 43, p < 0.01) (Fig. 2B).

Cold ratings did not differ between age groups after stimulation of the hand dorsum (W = 96, p = 0.35), hand palm (W = 123, p = 0.66), and foot (W = 83, p = 0.37).

### Test–retest reliability

**Figure 3:**
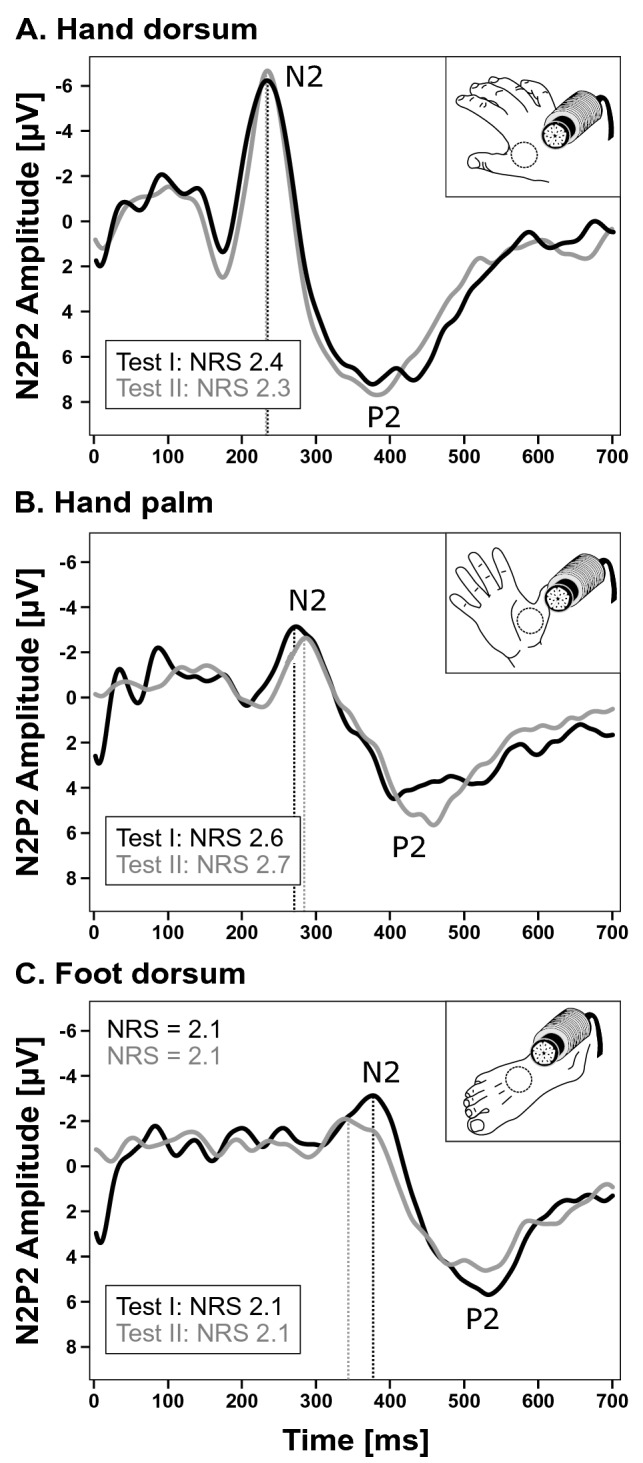
Grand averages of cold evoked potentials. Cold evoked potential negative–positive (N2P2) amplitudes of all individuals after stimulation of the hand dorsum **(A)**, hand palm **(B)**, and foot dorsum **(C)** at test I (black) and test II (grey). *NRS* numeric rating scale.

**Figure 4:**
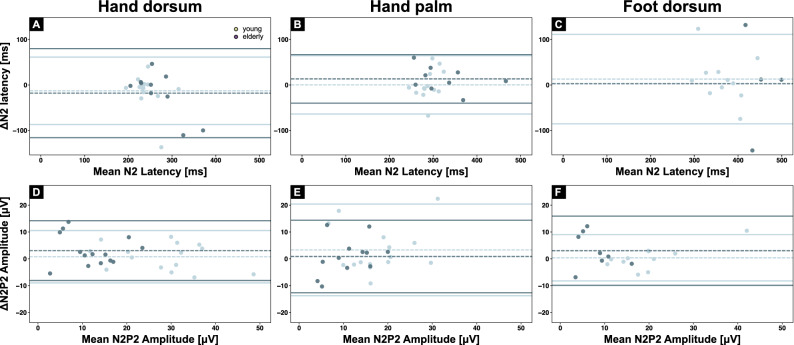
Reliability of cold evoked potentials. Bland–Altman plots for N2 latencies **(A–C)** and N2P2 amplitude **(D–E)** for all stimulation sites (i.e., hand dorsum, hand palm, and foot dorsum). Test–retest mean differences (dashed lines) and limits of agreements (solid lines) are shown for the young (light blue) and elderly age group.

The CEP grand averages at test I and test II of all individuals (young and elderly) and for all three testing sites are shown in Fig. 3. All test–retest analyses (ICCs and BA coefficients) are summarized in Table 1. BA plots for N2 latencies and N2P2 amplitudes for each stimulation site and for both age groups are shown in Fig. 4. Based on the ICCs, the young age group presented with “poor” to “fair” reliability for N2 latencies, “fair” to “substantial” reliability for N2P2 amplitudes. The elderly group presented with “fair” to “substantial” reliability for N2 latencies and “poor” to “fair” reliability for N2P2 amplitudes. Both age groups presented with “moderate” to “substantial” reliability for cold ratings.

## Discussion

The present study demonstrated improved feasibility and reliability of CEPs in response to rapid cooling of the skin (−300 °C/s) in healthy young individuals. CEPs could be recorded in all young individuals after stimulation of the hand, which was not achieved in our previous study using a slower cooling ramp (−20 °C/s)^20^. This methodological advantage allows the recording of CEPs from both the hand dorsum and palm, areas that are frequently affected by sensory deficits in patients with polyneuropathies and cervical myelopathies^7^. Herein lies a potential diagnostic opportunity, as CEPs can be used as a complimentary tool to LEPs and CHEPs to detect cold-specific deficits from both hairy and glabrous skin. The longer evoked potential latencies and increased amplitudes after stimulation of the hand palm compared to the hand dorsum have been described previously^20,25,26^. Thermal stimulus transduction is directly influenced by the thickness of the epidermal layer, which is increased in glabrous (i.e., thicker stratum corneum) compared to hairy skin^27^. In the present study we found shorter CEP latencies and increased amplitudes compared to previous studies applying slower cooling ramps (-20 °C/s)^20,26^. This is in line with recent studies also utilizing steeper cooling ramps^23^ and most likely driven by a more synchronous activation of cool-sensitive A-delta fibers achieved through steeper cooling ramps and larger temperature steps^22,28^. This in turn might explain the improved reliability of CEP parameters observed in the present study, in particular that of CEP latencies, compared to slower cooling ramps^20^. This is an important cornerstone for the integration of CEPs into the clinical neurophysiological routine, as response latencies are often considered a more robust characteristic of the afferent volley compared to the variability seen in amplitudes, which are more prone to modulatory effects due to attention and background noise^29^. Thus, the more synchronized afferent volley due to rapid cooling of the skin^22,28^ may lead to more robust signals and improved reliability of CEPs with faster latencies and larger amplitudes. In addition, the technical advances enable us to individually adapt the baseline to skin temperature. This in turn allows thermode displacement after each stimulus without the need to allow for appropriate adaptation (neutral perception), thus reducing potential habituation due to fatigue of peripheral cold receptors^30^ and most likely contributing to the improved reliability of CEPs^22^.

CEP latencies in response to rapid cooling of the foot dorsum were faster compared to a previous study adopting a slower cooling ramp^26^, which is similar to the aforementioned differences seen after stimulation of the hand presumably driven by higher responses in cool-sensitive A-fibers to rapid skin cooling^28,31^. In young individuals, CEP proportions were lower in response to rapid cooling of the foot (79%) compared to the hand with “fair” to “substantial” reliability of CEP parameters. Length and height dependent jitter are known to influence thermal sensitivity^32^ and signal dispersion of evoked potentials^16,33^. Longer peripheral conduction distances may yield less synchronized afferent volleys^34^ and thus lead to lower feasibility of CEPs in response to foot compared to hand stimulation. Moreover, reduced CEP amplitudes and longer latencies after stimulation of the feet could be explained by physiological reductions in ﻿intraepidermal nerve fiber density from rostral to caudal sites^35^ which are related to alterations of cortical latencies and amplitudes^36^. Although the hands are generally more sensitive to cold stimuli compared to the feet^32^, cold ratings did not differ between stimulation sites in the present study, which is in line with previous reports^23^ and most likely due to a floor effect and very low saliency of the stimulus (i.e., low cold ratings). Employing wider range rating scales from 0 (“not cold”) to 100 (“most imaginable cold”) may be more sensitive to detect differences between stimulation sites.

Overall, the elderly group presented with smaller CEP amplitudes and longer latencies compared to the young group, which is consistent with previous studies showing a substantial impact of age on other thermally-induced EPs such as CHEPs and LEPs^34,37^. Age-related alterations in thermal processing have been linked to subclinical changes in epidermal innervation^38^, mild neuronal loss, and dysfunction of peripheral nerves^32^. In addition, metabolic alterations in the skin of elderly individuals (i.e., reduced vascular supply) can hamper the functionality of cold-receptors as these are dependent on sufficient oxygen supply^39,40^. Nevertheless, rapid cooling of the skin led to relatively high CEP proportions in the elderly group (up to 82% for the hand dorsum) and “fair” to “substantial” reliability of amplitudes and latencies after stimulation of the hand. CEP recordings are thus feasible not only in younger, but also elderly individuals, which represents another important prerequisite for the implementation of CEPs into the diagnostic work-up of peripheral and central disorders presenting with alterations in cold processing predominantly present in elderly individuals.

In the present study, CEP acquisition after stimulation of the foot dorsum was particularly challenging in elderly individuals. These findings enunciate the notion that age-related alterations in thermal sensitivity are more pronounced in the feet compared to the hands^32,41^. The age-related vulnerability of long nerve fibers due to deceleration in axonal transport may lead to a more pronounced reduction in signal dispersion of the afferent volley in longer compared to shorter nerve fibers^42^. In combination with the lower epidermal nerve fiber density seen in elderly individuals^32^, this in turn may render the acquisition of CEPs after stimulation of the feet particularly challenging in elderly individuals.

To date, CEP alterations were reported in a handful of patients with cold allodynia and cold hypoesthesia due to different etiologies^15,18,23,26^. Based on the lower reproducibility in previous studies adopting slower cooling ramps, CEP alterations in patients with small-fiber neuropathy and central disorders need to be interpreted with caution, especially in elderly individuals due to the poor signal-to-noise ratio^20^. The improved feasibility and reliability of CEP recordings through rapid cooling of the skin will further expedite the clinical implementation of CEPs. In particular, recording of CEPs from the upper extremities in elderly patients with cold-specific deficits is feasible with rapid cooling of the skin. Indeed, dissociative findings between CEPs and LEPs (abolished CEPs and preserved LEPs) highlighted the use of CEP as a complimentary neurophysiological tool to assess cold-specific alterations in somatosensory function in elderly individuals with neuropathic pain^23^. These cold-specific alterations in peripheral neuropathies may be driven by damage of cool-sensitive primary afferents, likely expressing transient receptor potential channel melastin (TRPM8), the lack of which leads to loss of cold sensation in mice^43^. ﻿Cold allodynia can also be induced experimentally by topical application of menthol activating TRPM8, which leads to peripheral sensitization of cold-sensitive fibers^44^. Furthermore, cold-specific dorsal horn neurons and thalamic nuclei have also been reported in humans and may be differentially damaged and lead to dissociative findings in patients with central lesions affecting cold-specific fibers^17^. In patients with discomplete spinal cord injury, the development of neuropathic pain was associated with preservation of cold sensation and residual spinothalamic integrity^45^. In this sense, an objective neurophysiological tool to assess cold-specific fiber tracts would be extremely valuable. Further prospective studies are warranted to demonstrate the validity of multi-modal neurophysiological tools to assess the integrity of cold-specific pathways, preferably in comparison to heat-specific ones tested by CHEPs, in patients with cold allodynia and cold hypoesthesia.

The particularly challenging acquisition of CEPs from the lower extremities in elderly individuals is worth noting in a clinical context. In a previous study, CEPs were abolished after stimulating of both the affected and unaffected foot in an elderly post-stroke patient with cold allodynia^26^. This case illustrated the limitation of recording CEPs from the distal feet with previous methods (i.e., slow cooling ramps). Considering the results of this study, this remains a noteworthy limitation, even with newly developed faster cooling ramps. To overcome this issue, cold stimulators with larger stimulation surfaces may be required to improve the acquisition of CEPs from the feet in elderly patients, which may not be necessary for acquiring robust CEPs from the upper extremities. Enlarging the stimulation surface would result in spatial summation of afferent signals due to the simultaneous activation of a greater number of cutaneous primary afferents responsive to cold stimuli to counteract the lower density of cool-sensitive fibers in the feet^22^. These methodological concerns should be addressed in future investigations to enable the effective implementation of CEPs into the diagnostic work-up of patients with small fiber neuropathy, which initially manifests in the feet of elderly individuals. At the forefront, early diagnosis of small fiber pathology may disclose subsequent subclinical large fiber damage^46^ and is crucial for the timely administration of therapeutic interventions^47^.

Taken together, rapid cooling of the skin improved the feasibility and reliability of CEPs in young individuals. This elucidates the usefulness of CEPs as a complimentary objective neurophysiological tool to asses small fiber damage (i.e., cold-specific) and spinothalamic tract integrity in a wide range of neurological disorders. The recording of CEPs still remains challenging in elderly individuals, especially from the lower extremities (i.e., foot dorsum). Further experimental studies are warranted to establish favorable stimulation parameters (i.e., larger stimulation surfaces combined with steep cooling ramps and larger temperature steps) to increase the robustness of CEPs from all stimulation sites in elderly individuals. With these concerns in mind, future clinical studies are needed to validate the use of CEPs to detect alterations in cold-specific pathways in patients with neuropathic pain suffering predominantly form cold deficits and/or cold allodynia.

## Methods

### Individuals

The study included healthy individuals from a young (20–40 years) and elderly (50–70 years) age group. Exclusion criteria comprised current pain, any neurological condition, and intake of any medication (except for birth control). All procedures were in accordance with the Declaration of Helsinki and were approved by the local ethics boards (Kantonale Ethikkommission Zürich: EK-04/2006, PB_2016-02051). All individuals provided written informed consent prior to participating in the study.

### Study design

All individuals participated in two sessions (i.e., test I and test II), planned two weeks apart. Prior to both sessions, all individuals completed a general medical history questionnaire to detect any exclusion criteria missed upon initial recruitment. In order to exclude subclinical deterioration in somatosensory function, small and large fiber function was assessed semi-quantitatively by a bedside sensory exam including pinprick and light touch testing, respectively. In the elderly group, a neurologist (J.R.) performed an additional thorough clinical examination of the upper and lower extremities to exclude possible unknown age-related large and/or small fiber pathologies. This included the assessment of thermal sensation with a cold and warm thermoroller (Somedic SenseLab AB, Sweden), reflexes (i.e., knee and ankle jerks, biceps and triceps tendon reflex), muscle strength, and vibration detection with a tuning fork. Additionally, thermal quantitative sensory testing (QST) (i.e., cold detection and pain thresholds (CDT/CPT), warm detection/heat pain thresholds (WDT/HPT), and paradoxical heat sensations (PHS)) was performed at the hand and foot dorsum in the elderly individuals to exclude possible subclinical abnormalities. QST was performed using the standardized equipment and instructions provided by the German Research Network on Neuropathic Pain (DFNS)^48^. Briefly, thermal stimuli (baseline 32 °C; ramp 1 °C/s) were applied to the skin using a 30 × 30 mm ATS thermode (Pathway, Medoc, Israel). CDT, WDT, CPT, HPT, and PHS were determined using a response unit controlled by each individual. Prior to testing, the skin was heated to 32 °C with a warm water and the thermode was placed on the skin until the temperature was felt as neutral. The target skin temperature of 32 °C was assessed with a thermometer in all individuals.

### Cold stimulation paradigm

Rapid cold stimuli were applied to (1) the hand dorsum (i.e., hairy skin), (2) the hand palm (i.e., glabrous skin), and (3) the foot dorsum. The stimulation sites were chosen to assess rapid cooling at distal sites of upper and lower extremities which are most commonly affected areas in patients with small fiber neuropathies^6,12^ and cervical myelopathy^49^. The order of testing (i.e., stimulation sites and body side) was randomized between individuals. The transcutaneous thermal stimulator (TCS) (T03 probe, QST.Lab, Strasbourg, France) has a 1.2 cm^2^ total surface area, consisting of 5 stimulation zones (3 × 3.2 × 2.4 mm) and each zone contains 3 micro-Peltier elements. The TCS thermode was calibrated to the skin temperature of each stimulation site prior to each measurement. If the skin temperature was below 30 °C, the testing site was heated using a warm water bath before proceeding with the assessments. The cooling ramp was fixed to −300 °C/s and the destination temperature was set to 20 °C below skin temperature. Fifteen rapid cold stimuli (1500 ms) were applied to the stimulation sites with a random interstimulus interval of 8–12 s. This stimulation paradigm allows recording CEPs with a robust signal-to-noise ratio^22^ and represents a feasible clinical implementation in terms of time expenditure. The probe was repositioned after each stimulus to minimize peripheral receptor fatigue and habituation^30^. Four seconds after stimulus onset, individuals were cued by a tone/beep to rate the perceived coldness intensity on a numeric rating scale (NRS) from 0 (i.e., not cold) to 10 (i.e., most imaginable cold).

### Cold evoked potential recording setup

For the recording of CEPs, all individuals were in a supine position and asked to fixate a point on the ceiling and relax the facial muscles to reduce ocular and movement artefacts. A familiarization procedure was performed prior to the recording of CEPs, which consisted of five cold stimuli applied to the hand dorsum contralateral to the testing side. Cortical responses were recorded with 9 mm Ag/AgCl cup electrodes filled with conductive adhesive gel (Nihon Kohden, Tokyo, Japan) placed on the vertex (active—Cz) and both earlobes (references—A1/A2). The recording sites were prepared with Nuprep (D.O. Weaver & Co., Aurora, Co, USA) and alcohol. Electrooculographic (EOG) signals were recorded from surface electrodes (Ambu^®^ BlueSensor NF, Ballerup, Denmark) placed above and below one eye for online monitoring of potential signal artefacts due to ocular movements and eye blinks. All signals were sampled at 2000 Hz with a preamplifier (20,000×, ALEA Solutions, Zurich, Switzerland) and bandpass-filtered between 0.5 and 30 Hz. The recording window was set to 500 ms pre-trigger and 1 s post-trigger in a customized program based on LabView (V2.6.1. CHEP, ALEA30 Solutions, Zurich, Switzerland).

### Data analysis and statistics

CEP signals contaminated with eye blinks were excluded online and additional cold stimuli were applied to generate an averaged evoked potential of 15 artefact free signals per stimulation site. The N2 latency and N2P2 waveform, detected as the peak-to-peak amplitude, were identified from the averaged evoked potential by three independent raters (P.S., N.N., M.H.). Offset correction based on the 500 ms pre-stimulus window was employed on the averaged CEP signals prior to statistical analyses.

Statistical analyses were conducted using RStudio statistical software for Windows, version 3.5.3. All data was tested for normal distribution by visual inspection of histograms and QQ-plots and statistical tests were chosen based on data distribution (i.e., non-parametric tests for non-normally distributed data). Statistical significance was set at α = 0.05.

Differences in CEP parameters (i.e., N2P2 amplitude, N2 latency, cold ratings) between sites (i.e., hand dorsum, hand palm, and foot dorsum) were tested with the the Skillings-Mack test with post-hoc analyses (i.e., Bonferroni correction for multiple comparisons). Differences in CEP parameters between age groups (i.e., young and elderly) were tested with the Mann–Whitney test.

Test–retest statistics were performed for all CEP parameters using intraclass correlation coefficients (ICC, two-way mixed, single measure) and Bland–Altman (BA) analysis. ICC values were defined as “poor” (< 0.40), “fair” (0.41–0.60), “moderate” (0.61–0.80), and “substantial” (0.81–1.00)^50^. BA plots were employed to estimate the limits of agreement between test I and II for CEP latencies and amplitudes. Individuals with absent CEPs (N2P2 amplitude = 0) at both test I and II were excluded from reliability analysis of N2P2 amplitudes. Individuals with absent CEP at test I, test II, or both were excluded from reliability analysis of N2 latencies.
